# A new method using pointing area diagrams to characterize and compare ventilation masks used in continuous positive airway pressure treatment for apneic patients

**DOI:** 10.1007/s00405-025-09539-x

**Published:** 2025-07-18

**Authors:** Baptiste Rouchié, Maxime Fieux, Steeve Reisberg, Yann Retory, Alexandra Schmidt, Benoit Piro, Giorgio Mattana, Marcel Filoche, Bruno Louis, Clara Virbel-Fleischman, Emilie Béquignon

**Affiliations:** 1 Explor Center, Air Liquide SA, Bagneux, Île-de-France France; 2 ITODYS, CNRS UMR 7086, Université Paris Cité, Paris, Île-de-France France; 3 Hospices Civils de Lyon, Centre Hospitalier Lyon Sud, Service d’ORL, Lyon, Auvergne-Rhône-Alpes France; 4 Université Lyon 1, Université de Lyon, 69003 Lyon, Auvergne-Rhône-Alpes France; 5 Ecole de Médecine, Université Paris-Est Créteil (UPEC), Créteil, Île-de-France France; 6 IMRB, INSERM, Université Paris-Est Créteil (UPEC), 94010 Créteil, Île-de-France France; 7 CNRS EMR 7000, 94010 Créteil, Île-de-France France; 8 Service d’ORL Et de Chirurgie Cervico-Faciale, Centre Hospitalier Intercommunal de Créteil, Créteil, Île-de-France France; 9 Service d’ORL, d’otoneurochirurgie Et de Chirurgie Cervico-Faciale, Centre Hospitalier Lyon Sud, Hospices Civils de Lyon, 69310 Lyon, Pierre-Bénite France

**Keywords:** OSA, Masks, Interfaces, CPAP, Comparison, Cutaneous pressure, Unintentional leaks, Adherence, Pointing area diagrams

## Abstract

**Purpose:**

Mask choice is a key parameter in the adaptation of continuous positive airways pressure (CPAP) treatment. Two indicators used to evaluate poor mask tolerance are cutaneous overpressure and unintentional leaks. The main aim of this study was to characterize each mask, thanks to a feedback harvesting method using pointing area diagrams.

**Methods:**

Diagrams showing a face scheme were submitted to 70 health professionals who install masks. They pointed out the areas of cutaneous overpressure and of unintentional leaks for 6 different masks (2 of each type: facial, nasal, pillow). Areas on the face with the highest concentration of points were determined to compare masks, regarding pressure and leak points.

**Results:**

Out of the 396 analyzed diagrams, the nasal bridge was the area with highest pressure points concentration: 33% and 30%, for facial and nasal masks, respectively. Internal canthus was the area with highest leak points concentration: respectively 27% and 41%. On the nasal bridge, there was no significant difference between facial and nasal masks regarding pressure points (74%, 76%, 72%, and 63%). On internal canthus, 31% indicated a leak point for the F20, 40% for the Quattro Air without significant difference whereas the report was increased for the Soft nasal in comparison to the other nasal mask the Mirage FX (61% vs 33% respectively, *p* < 0.05).

**Conclusion:**

This method could help decision-making of physicians, health professionals and could be useful for manufacturers in the improvement of their products.

**Trial registration number:**

#20240510.

## Introduction

Obstructive Sleep Apnea Syndrome (OSAS) is a frequent breathing disorder, which has seen a world-wide increase on its prevalence. It was estimated that in 2019, more than 1 billion people were affected by OSAS worldwide (adults with mild to severe obstructive sleep apnoea) [1]. This pathology is associated with cardiovascular comorbidities and daytime sleepiness [2–4]. Continuous Positive Airway Pressure (CPAP) treatment is nowadays considered as the gold standard to treat OSAS symptoms in an efficient manner [5, 6]. Nevertheless, the biggest reported disadvantage of CPAP is the poor patient adherence. Indeed, and depending on the used method, as well as the study duration and localization, the adherence rate can go from 40% up to 80% [7–10]. Recently, Pépin et al*.* presented a study on 480 000 patients, which demonstrated that the treatment termination increases with time, from 23% of termination rate after 1 year of therapy, and up to 48% after 3 years of therapy [10]. Moreover, since CPAP is a palliative treatment that only treats the symptoms [11], the adherence is a crucial stake in the therapy [12].

Although patient adherence is linked to multiple factors, such as his/her environment, the disease, and the treatment [13–16], one of the major factors is the adaptation of the ventilation interface (*i.e.* the mask) to the patient's face morphology [15]. In fact, this mask is the device that is positioned in the interface between the CPAP machine and the patient’s face. It plays the role of pneumatic seal, since it has to adapt to the patient's face morphology, at the same time it ensures the delivery of the therapeutic pressure. For these reasons, two indicators that are used to evaluate the mask adaptation can be described: the cutaneous overpressure [17] and the unintentional leak [18]. Mask adaptation is such a stake that mask customization through additive manufacturing can be considered in order to reduce overpressure and leaks risk [19, 20]. Nonetheless, to the best of our knowledge, there is no study that describes a method to compare different masks of the same type among themselves.

The main purpose of this study was to understand the most common pressure points and unintentional leak points in CPAP treatment according to mask type. Each mask was characterized through an exploratory study by using a feedback harvesting method through pointing area diagrams that are proposed to Health Professionals (HP) responsible for installing masks at patient homes.

## Methods

### Design of the study

**Fig. 1:**
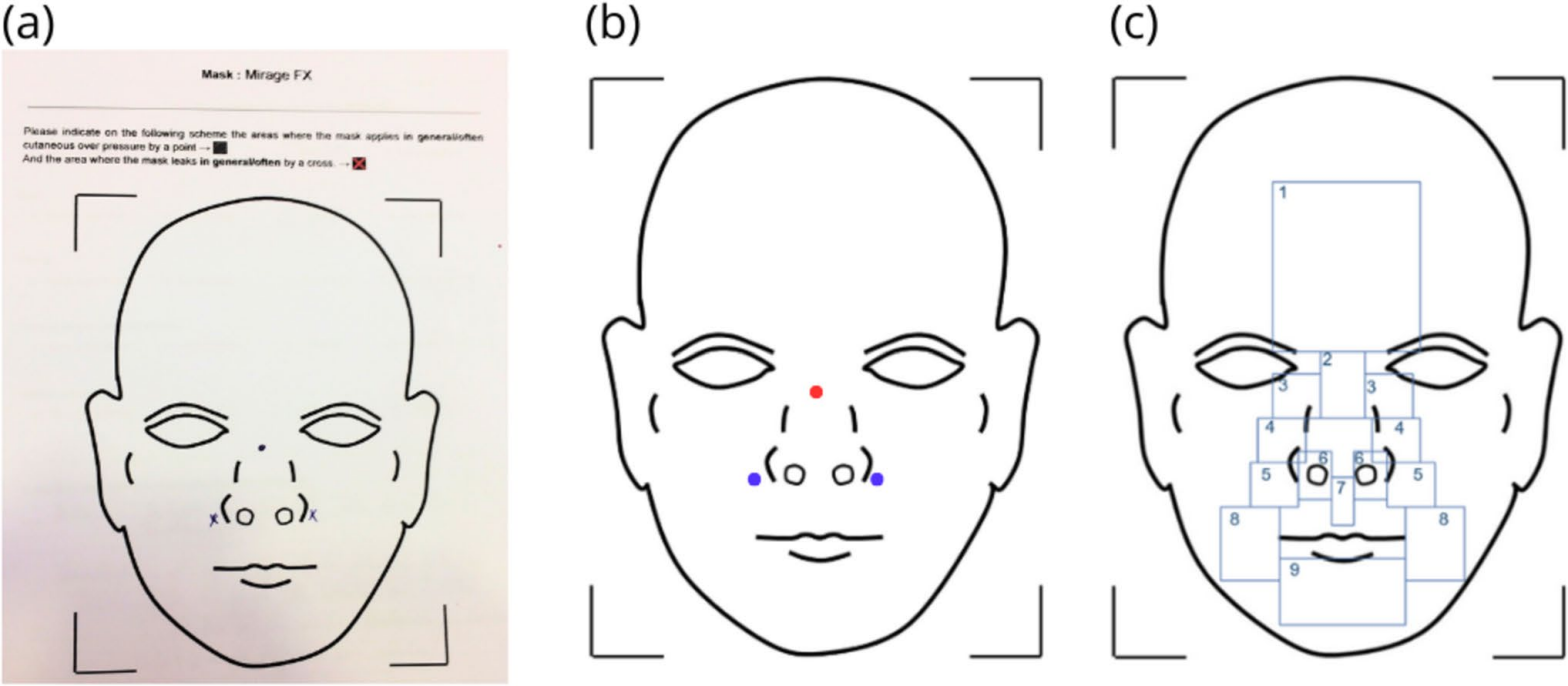
Example of data collection and treatment (**a**) Example of the scanned answer of HP#2, In here, one HP indicated that, according to his experience, the Mirage FX leaks often on the side of the nose, applying too much pressure on the nasal bridge of patients. (**b**) Post-treated points of both pressure and leak, indicated by one of the HP that reported on the original diagram (**c**) Original diagram divided in: 1-Forehead, 2-Nasal bridge, 3-Internal canthus, 4-Nosewings, 5-Outsides of the nostrils, 6-Nostrils, 7-Philtrum, 8- Cheeks, 9-Chin

This was a monocentric, prospective observational study including volunteers HPs from one Home Medical Equipment Provider (Vitalaire, Portugal) who were asked to fill a pointing area diagram for 6 different masks. This study was conducted according to the Declaration of Helsinki (WMA, 1997) and was approved by local ethics Committee (approval number: 2024–05-10, *Comité d’éthique local Recherche du CHIC,* Creteil, France). All HPs participating in the study were informed of the modalities and interest of the protocol through an information note joined to the questionnaire. Only volunteers HPs sent back the document, thus the need for writing consent was waived by the ethics committee. HPs had a median practicing experience of 6 years [1-19 years] and came from various regions in Portugal. The test was designed for more than 50 participants, *i.e.* the number to provide sufficient variations in exploratory studies [21]. This survey was designed considering Standards for Reporting Qualitative Research (SRQR) recommendations [22]. Masks were the two most installed masks of each type (facial, nasal, pillow) by Home Medical Equipment Provider. The facial masks were: Resmed AirFit F20 (Resmed, San Diego, United States), Resmed Quattro Air, the nasal masks were: Air Liquide Medical System (ALMS) Respireo Soft nasal (ALMS, Antony, France), Resmed Mirage FX, and the pillow masks were: ALMS Respireo Primo P, and Resmed Swift FX. It was noticed that the Resmed Quattro Air, the Respireo Soft nasal and the Resmed Mirage FX embed a frontal frame whereas the other masks do not. This Home Medical Equipment Provider does not provide only Resmed or ALMS masks, nonetheless there were the most bought masks. On the diagram, HPs were asked to point out, according to their experience, the area(s) where the masks were more likely to apply cutaneous overpressure (with a dot) or else to leak (with a cross). Afterwards, they scanned and anonymously sent back their answers, as shown with the example presented in Fig. 1(a).

### Data treatment

The first step was to determine the areas on the face with highest concentration of points (pressure and leaks), according to the mask type: i. e., key areas. The second step was to determine the frequencies of answered scenarios given by HP, between areas both with highest pressure point concentration and with highest leak point concentration. Finally, the last action was to compare masks of the same type, regarding pressure and/or leak points, in the previously described areas.

For each scanned diagram, the number of dots and crosses were counted according to each mask. This allowed to obtain the number of pressure and leak points per HP, for each mask. Then, the coordinates of dots and crosses were gathered, in regards to the original diagram. For instance, as exposed in Fig. 1, HP’s indicated points for each mask (a) were identified and reported on the original diagram (b), and then the original diagram was divided into 9 areas: Forehead, Nasal bridge, Internal canthus, Cheeks, Chin, Nosewings, outside of the Nostrils, Nostrils, Philtrum (c). These areas were chosen considering geometrical reasons, as they represent tangent plans of the face and all points located in each zone by HP are part of one 2D plan. Each point reported in those areas was classified according to its coordinates on the original diagram. All the points that were positioned out of the 9 areas were assigned to an area named “others”. In this example, the points indicated by HP#2 shown in Fig. 1(a) were sorted in the areas 2 and 5, respectively nasal bridge and outside of the nostrils. We obtained the repartition of points per area according to mask type, *i.e.* the number of points in each area relatively to all points indicated by all the HPs.

Then, and by using the total repartition of points for each type of mask, the relationship between the area with the highest concentration of pressure points, as well as the area with the highest concentration of leak points were studied. In each area, the possible HP answers scenarios where the following can be considered: no point, one (or more) pressure point, one (or more) leak point, one leak point and one pressure point. The frequency of each possible scenario in the area of highest-pressure concentration was studied according to the frequency of each possible scenario in the area of highest leak concentration.

Finally, all the areas with a point concentration value of more than 5% were considered as key areas for each type of mask. For each mask, the number of HP that indicated one (or more) pressure or leak point in each key area was determined. This allowed to obtain the proportion of HP who indicated points (pressure or leak) in the key areas of facial masks for the F20 and the Quattro Air, in the key areas of nasal masks for the Soft Nasal and the Mirage FX, and in key areas of the pillow masks for the Swift FX and the Primo P.

### Statistical analysis

Categorical variables were expressed as frequencies and percentages, being compared between groups using Chi square tests (or Fisher's exact test when the former was not applicable). Continuous variables were summarized as means and standard deviations, having first been voted and were compared between groups using Student's t test (or Wilcoxon tests if the former was not applicable). The threshold for statistical significance was 0.05. All statistical analyses were performed with R software (v. 4.1.2, R Foundation for Statistical Computing, Vienna, Austria, www.r-project.org).

## Results

### Feedback and point countdown per mask and HP

Seventy HP were enrolled for this study and the rate of response was 100% (70/70). Three-hundred-ninety-six diagrams filled by HP were collected: 70 for the F20, 68 for the Quattro Air and the Soft Nasal, 64 for the Primo P, and the Mirage FX; and 62 for the Swift FX. HP indicated 2.1 ± 1.9 [0; 17] (mean ± standard deviation [range]) points of pressure per mask and 2.4 ± 1.4 [0; 8] points of leaks per mask. Regarding the types of masks, HP indicated less pressure points for pillow masks compared to other masks: 1.7 ± 1.6 against 2.3 ± 2.2 for facial and 2.3 ± 1.8 for nasal (*p* < 0.05). They indicated 2.6 ± 1.4 leak points for facial masks, 2.3 ± 1.5 leak points for nasal masks, and 2.2 ± 1.3 leak points for pillow masks (no statistical difference, *p* > 0.1).

### Localization and repartition in areas of points according to the mask type

**Fig. 2:**
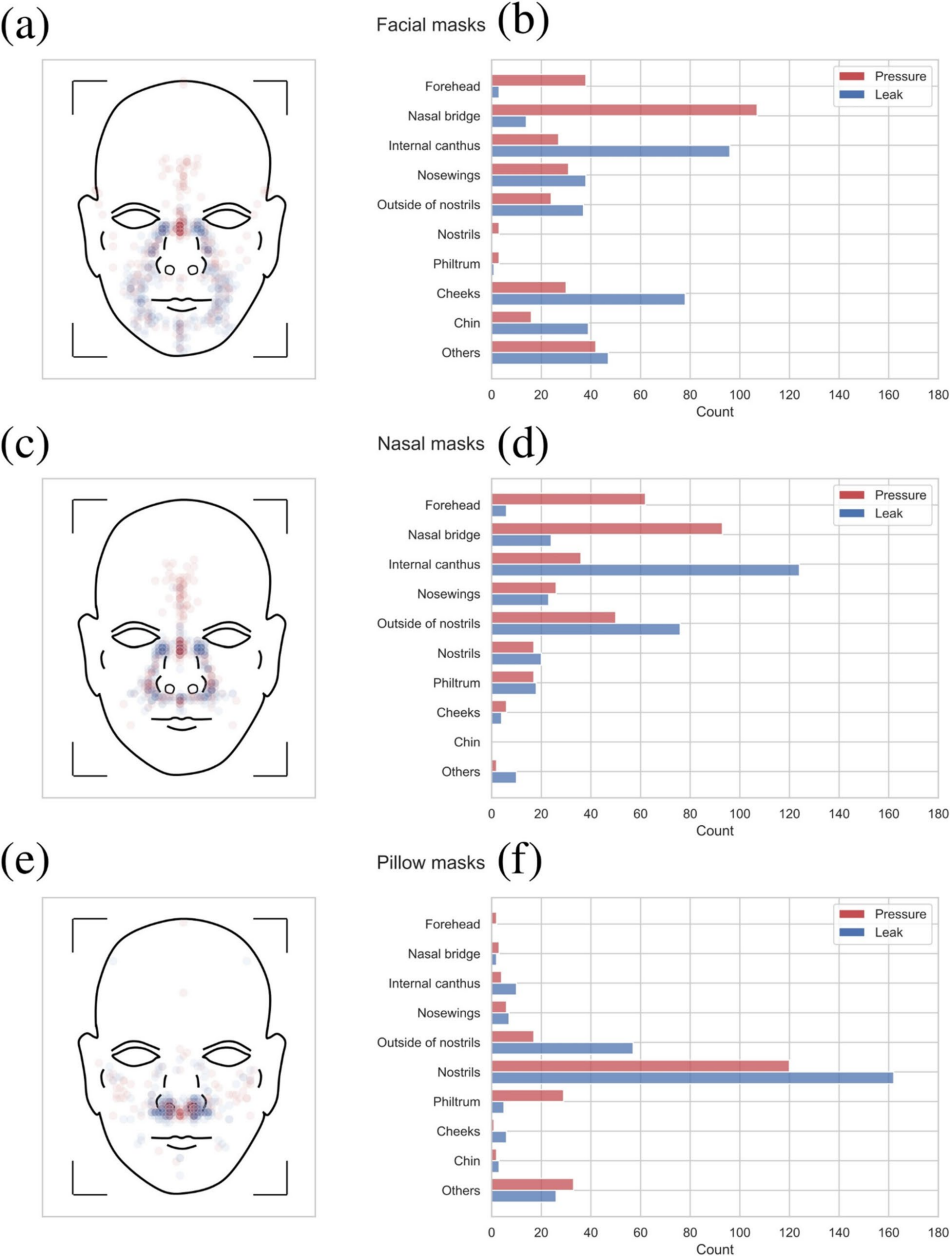
Point repartition per mask type. Pressure (red) and leak (blue) points reported on the original diagram for facial (**a**), nasal (**c**) and pillow masks (**e**). Number of pressure (red) and leak (blue) points per area for facial (**b**), nasal (**d**) and pillow masks (**f**)

On diagrams, 1783 points were analyzed, with a total of 847 pressure points and 936 leak points. Ninety-one percent of the points (*n* = 1621) were located in the nine areas. The use of facial masks gathered 321 pressure points and 353 leak points. In the case of nasal masks, 309 pressure points and 305 leak points were indicated. For pillow masks, 217 pressure points and 278 leak points were considered. The repartition of points according to each type of mask for areas and especially the key areas with the 5% threshold are shown in Fig. 2. The four HP (6%) indicating pressure points on the forehead for the F20 might have been inaccuracies due to scheme definition.

Facial and nasal masks shared the same area with the maximum pressure and leak points concentration: nasal bridge for pressure and internal canthus for leaks. In fact, the nasal bridge pressure points concentration was 33% (*n* = 107) for facial masks and 30% (*n* = 92) for nasal masks. Regarding the internal canthus, the concentration of leak points was 27% (*n* = 96) for facial masks and 41% (*n* = 124) for nasal masks. Also, for facial and nasal masks, there were more than 7% pressure and leak points on the internal canthus, in the nosewing area and outside of the nostrils (Fig. 2(b), (d)). The forehead area saw a concentration of 12% (*n* = 38) of pressure points for facial masks, and 20% (*n* = 62) for nasal masks. For pillow masks, the area with the maximum concentration of pressure, 55% (*n* = 120), and of leak points, 58% (*n* = 162), was the nostrils area. Otherwise, outside of the nostrils concentrated 21% (*n* = 57) of leak points and 8% (*n* = 17) of pressure points. The philtrum area gathered 13% (*n* = 29) of the pressure points.

Here, it was considered that the forehead, nasal bridge, internal canthus, nosewings, outside of the nostrils, cheeks and chin were contact areas for facial masks. Furthermore, the forehead, nasal bridge, internal canthus, nosewings, outside of the nostrils, philtrum and nostrils were contact areas for nasal masks. And that contact areas for pillow masks were outside of nostrils, nostrils and philtrum. When looking at the repartition of points, 85% (*n* = 273/321) of pressure points were located on the contact areas for facial masks, 97% (*n* = 301/309) for nasal ones, and 77% (*n* = 166/217) for pillow ones (Fig. 2). For instance, as shown in Fig. 2, the seal contact areas were highly represented, especially internal canthus for facial and nasal masks with respectively 27% (*n* = 96/353) and 41% (n = 124/305), and nostrils for pillow masks with 58% (*n* = 162/278) of the points.

### Scenario frequencies in high concentration areas

**Fig. 3:**
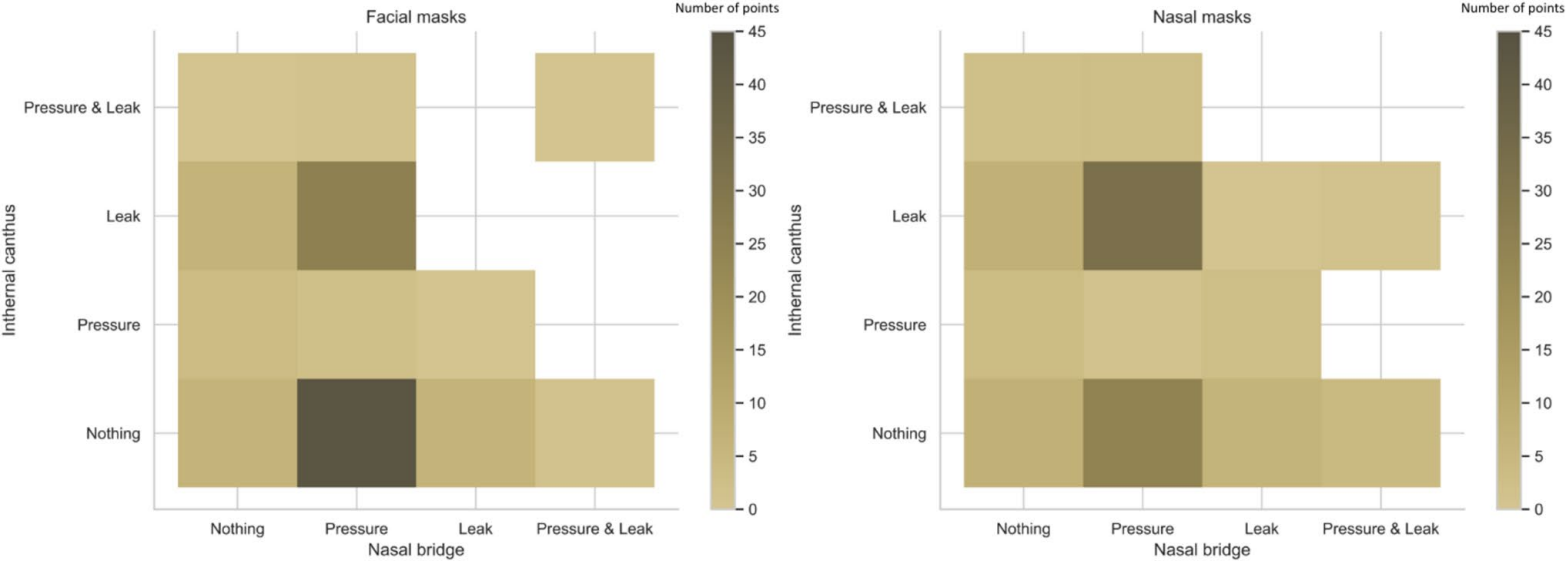
Frequencies of answered scenarios given by HP concerning pressure and leak in highest concentration areas determined above: the nasal bridge and the internal canthus for facial masks (**a**) and nasal masks (**b**). The shade scale indicates the frequency of scenarios in percentage (the darker is the area the more frequent is the scenario)

In a second step, a study regarding the frequencies of possible HP answered scenarios in the highest leak concentration areas, according to the possible HP answers in the highest-pressure concentration areas, was performed for each type of mask. With this, it was possible to determine the frequency of each possible answer combination (scenario) in those two areas (Fig. 3). For facial masks, in 43% of the cases (*n* = 60/138), HPs indicated pressure points on the nasal bridge and no point on the internal canthus, as shown in Fig. 3(a). When HPs indicated a leak point on the internal canthus, they indicated a pressure point on the nasal bridge in 80% (*n* = 36/45) of the cases. For nasal masks, as shown in Fig. 3(b), HPs indicated pressure on the nasal bridge and leak in the internal canthus area in 33% (*n* = 43/132) of the cases. Regarding pillow masks, the most concentrated area was the nostrils for both pressure and leak points. It was noticed that in 37% (*n* = 46/126) of the cases, HPs indicated a leak point without pressure point, in 23% (*n* = 29/126) of the cases HPs indicated a pressure and a leak point, and in 60% (*n* = 75/126) of cases a leak point was indicated.

### Proportion of points per type of mask in key areas

The number of HPs who indicated a point (pressure or leak) in each key area is shown in Table 1. In relation to the pressure points of facial masks, 74% of HPs indicated a pressure point on the nasal bridge for the F20, and 76% for the Quattro Air, which is not a significant difference. Pressure was indicated on the forehead by 6% of HPs for the F20, and 40% of them for the Quattro Air, with a significant difference (*p* < 0.001). Concerning the leak points of facial masks, the report of leaks by HPs on the internal canthus was decreased in the F20, when compared to the Quattro Air (31% versus 40%, respectively), without a significant difference, which is in the same line with the other nine anatomical areas.

**Table 1:** HPs responses for each type of mask, divided into their key areas

Facial masks	Areas	Number of HP (percentage)		*p* value
		F20, *n* = 70	Quattro Air, *n* = 68	
Pressure	Forehead Nasal bridge Internal canthus Nosewings Outside of the nostrils Cheeks	4 (6%) 52 (74%) 8 (11%) 7 (10%) 5 (7%) 10 (14%)	27 (40%) 52 (76%) 5 (7%) 11 (16%) 8 (11%) 6 (9%)	< 0.01* 0.92 0.60 0.41 0.52 0.46
Leak	Internal canthus Nosewings Outside of the nostrils Cheeks Chin	22 (31%) 10 (14%) 7 (10%) 19 (27%) 17 (24%)	27 (40%) 9 (13%) 13(19%) 22 (32%) 13 (19%)	0.40 1 0.20 0.63 0.60
Nasal masks	Areas	Number of HP (percentage)		
		Soft Nasal, *n* = 68	Mirage FX, *n* = 64	
Pressure	Forehead Nasal bridge Internal canthus Nosewings Outside of the nostrils Nostrils Philtrum	29 (42%) 49 (72%) 8 (11%) 7 (10%) 17 (25%) 8 (11%) 9 (13%)	20 (31%) 40 (63%) 10 (16%) 7 (11%) 10 (16%) 1 (2%) 8 (13%)	0.24 0.32 0.70 1 0.26 0.03* 1
Leak	Nasal bridge Internal canthus Nosewings Outside of the nostrils Nostrils Philtrum	14 (21%) 42 (62%) 9 (13%) 18 (26%) 7 (10%) 10 (15%)	7 (11%) 21 (33%) 4 (6%) 22 (34%) 5 (8%) 8 (13%)	0.20 < 0.01* 0.30 0.43 0.85 0.91
Pillow masks	Areas	Number of HP (percentage)		
		Primo P, *n* = 64	Swift FX, *n* = 62	
Pressure	Outside of the nostrils Nostrils Philtrum	3 (5%) 29 (45%) 19 (30%)	7 (11%) 28 (44%) 10 (16%)	0.20 0.98 0.10
Leak	Outside of the nostrils Nostrils	13 (20%) 35 (55%)	17 (27%) 40 (64%)	0.47 0.35

The shown numbers correspond to the absolute (and relative) values for the categorical variables. ^*^ indicates statistical significance, *p*-value < 0.05

Concerning nasal masks, 72% of HPs indicated a pressure point for the Soft Nasal, and 63% for the Mirage FX, without a significant difference. There were respectively 42% and 31% of HPs that indicated pressure on the forehead for the Soft Nasal and Mirage FX, without a significant difference. The only area that showed a significant difference of pressure between the Soft Nasal and Mirage FX was the nostrils (2% versus 8%, respectively, *p* < 0.05). The report of leak points on the internal canthus was significantly increased for the Soft Nasal, when compared with the Mirage FX (61% versus 33%, respectively).

Finally, for pillow masks, the nostrils zone was a key area for pressure and leak points. Pressure points were reported on nostrils by 45% of HPs for the Primo P and by 44% for the Swift FX. Leaks were also reported on nostrils by 55% and 64% of HPs for the Primo P, and the Swift FX, respectively.

## Discussion

This exploratory study helped us understand the most common pressure points (nasal bridge, nostrils) and unintentional leak points (internal canthus, nostrils) of masks in CPAP treatment and provides a new method to characterize and compare masks by using pointing area diagrams. It brings new insights on the localization of mask non-adaptation indicators, *i.e. *cutaneous overpressure and unintentional leaks, leading clinicians towards a tailored approach and personalized medicine.

It was shown that nasal masks show a higher efficiency in order to keep open airways, since facial masks apply pressure on the chin leading to a narrowing of the airways and usage of nasal masks ensures the natural nasal breathing [23, 24]. Furthermore, it has been demonstrated that using nasal masks allows reaching a better adherence to treatment, a higher number of treatment hours, a lower therapeutic pressure, and even a lower residual Hypopnea Apnea Index (HAI) when compared with facial masks.[15, 25–27] This resulted in the recommendation of the American Academy of Sleep Medicine [28] and from the French Language Pneumology Society (SPLF) [29] to use nasal masks as a first choice. Regarding pillow masks, no clinical aspects were demonstrated to be different when compared to nasal masks [30]. No studies were found that described a method to compare different masks of the same type among themselves. This lack could be explained by the fact that mask release frequency is higher than the required time for these kinds of investigations. The other aspect that can explain this lack of studies is the fact that patient adherence is multifactorial, so studying the only impact of the mask is biased. Regarding this, an easy way to characterize and compare same type masks was proposed.

We developed and used a questionnaire dedicated to the evaluation of HPs experience on pressure and leaks of masks. Questionnaires for measuring daytime sleepiness [31] or usability [32] and user experience [33] exist. These questionnaires were used to rate treatment efficiency [34] or to characterize medical devices [35]. However, they do not allow to specifically localize and quantify the main issues (pressure and leak) due to mask adaptation. The proposed pointing area diagram was created to quantify those main issues. In addition, it was conceived to be an intuitive survey so that HPs could easily and quickly answer. Those questionnaires could be proposed to other HPs from other countries but also to patients, especially the ones experiencing trouble in finding a mask adapted to their morphology. Likewise, they could be used with other masks, in order to create a large comparison database of the masks to propose the most suited masks to every patient.

In this study, the number of leak points was not different between facial and nasal masks (respectively, 2.6 ± 1.4 against 2.3 ± 1.5, *p* > 0.05). This result was not concordant with Rowland et al*.,* which described a longer CPAP time with a large leak with facial masks over nasal ones [26]. Nevertheless, the pointing area diagram gives information of the leaks localization but not their intensity nor their flow. Therefore, for the same number of leak points of a facial and a nasal mask, the unintentional leakage flow could be higher with facial masks. The number of leak points for pillow masks (2.2 ± 1.3) were not significantly different from the number of other mask types, which follows the Zhu et al*.* study [36]. Indeed, they compared to pillow masks, having not observed any differences between 95th percentile leak level even, for PAP level above 15 cmH_2_O. There were significantly less pressure points for pillow masks, when compared to other masks. However, in studies based on qualitative data [36, 37], pillow masks were reported to be less stable on patient faces. This shows that probably pillow masks'minimalist design allows the avoidance of pressure on large areas of patients’ faces, while in return the compromise is that those designs could lead to stability issues.

Localization of pressure applied by masks was investigated through the presence of ulcers [38]. In their study, Schallom et al. observed that with facial masks used for noninvasive ventilation, ulcers were located on the different areas of patient face in contact with the mask and especially on the Nasal bridge with 17 ulcers out of 24 on this area. This is coherent with the proposed study, which showed that the majority of pressure points was located in contact areas. Nasal bridge area was the most represented area in Schallom et al*.* study, with the detection of 17 ulcers in this area, out of 24, while in our study 33% (*n* = 107/321) of the pressure points were in this area. Furthermore, in our case pressure points could be localized very quickly without reaching serious injuries on patient’s faces.

Valentin et al*.* showed that leaks, when adjusted to therapeutic pressure, were associated with a poor adherence: 7.0 ± 3.5 L/min/cmH_2_O for non-adherent patients, against 4.9 ± 1.7 L/min/cmH_2_O for adherent patient (*p* < 0.0001) [18]. Similarly, Rowland et al*.* used the CPAP time with large leaks to compare mask types [26]. They highlighted the importance of leak management, although they did not give any information on the leak localization (unlike the ones we could obtain thanks to HPs feedback). For instance, as shown in Fig. 2, the seal contact areas show a high representation, especially internal canthus for facial and nasal masks with one third of the points and nostrils for pillow masks, with two third of the points.

In our study, the focus was on the most critical areas of pressure and leak: the areas with the highest concentration of points (pressure and leak) per type of mask (Fig. 3). For facial masks, the most frequent scenario was a pressure point indicated on the nasal bridge, and nothing on the internal canthus. Nevertheless, when a leak point is indicated on the internal canthus, there is an indication of a pressure point on the nasal bridge in 80% (*n* = 36/45) of the cases (Fig. 3). Therefore, it can be suggested that facial masks leak on the internal canthus is the major cause of pressure on the nasal bridge, although this is not reciprocal. For nasal masks, the most frequent scenario is a pressure point indicated on the nasal bridge, and a leak point on the internal canthus. For pillow masks, since in 60% (*n* = 75/126) of the cases a leak point is indicated on the nostrils by HPs, it can be said that for those masks the nostrils adaptation is the most complicated stake. Pressure on the nasal bridge and leak on the internal canthus were the most reported events by HPs for facial and nasal masks (Fig. 3), which highlighted the fact that adaptation in those areas is a major stake in mask fitting. This has a higher importance considering that nasal bridge injuries and sore eyes are frequently reported issues of the treatment [39]. This could be explained by the morphologic complexity and variety of those areas. Consequently, a mask with a significantly smaller proportion of points per HP in those areas would have arguments to be a first installation choice.

Finally, masks of the same type can be compared, by looking at HPs responses in key areas. Obviously, it was reported a significantly higher pressure on the forehead for Quattro Air than for F20, since the Quattro Air has a frontal frame that does not exist in the F20. However, the Soft Nasal and Mirage FX have a similar design but the percentage of HPs who indicated leak on the internal canthus is significantly higher for the Soft Nasal than for the Mirage FX: 62% vs 33%, respectively. Therefore, according to HPs, it is more likely that a patient suffers from unintentional leaks on her/his internal canthus with the Soft Nasal than with a Mirage FX. Nonetheless, if a patient complains about either too much pressure in this area or leaks outside of the nostrils, switching from Soft Nasal to Mirage FX might be inefficient as there is no significant difference in those cases. Based on those results it was possible to identify areas on which one of the pillow masks had an advantage (lower HPs responses) or a disadvantage (higher HPs responses) compared to another one. In order to propose best-fitted masks, custom-made masks can be designed from users'3D scan [19, 20]. In those studies, quantitative (force sensors, thermal detection of leak) and qualitative (user preferences, facial marking) values were gathered. It is noteworthy that the aim was to compare the developed prototypes to the commercial masks, while the comparison criteria were quantification and localization of force (*i.e.*pressure), as well as unintentional leaks. This showed that our survey could be beneficial and complementary for further mask development. Indeed, these results could be used to choose the best mask for each patient in a tailored approach based on specific morphometrics parameters identified herein. For instance, a patient with a thin nasal bridge would be more likely to accept a mask with fewer indicated pressure points on his nasal bridge.

### Strengths and weaknesses

A limit of this study is that HPs are not users of masks. It is possible that their interpretation of pressure and leak points on patients’ faces was biased by their experience, especially since they are from the same company, having the same mask selection. Another study with the same methodology could be interesting with users of CPAP, to quantify their judgment and experiences too, and even to compare it with HPs views. However, using patients could be biased also, reducing the interrater reliability and introducing a cognitive bias. Finally, it is possible that this method is not accurate enough for pillow masks comparison. Indeed, it was observed that for pillow masks less key areas were indicated, with points being mainly in the nostrils area. To cope with this last limitation, a more detailed scheme of the nasal area could be provided to harvest more data in smaller areas, such as nose tip, columella, and vestibule. Nevertheless, this original exploratory study allowed us to quantify HPs user experience for each mask eventhough only one Home Medical Equipment Provider was chosen. Indeed, through HPs'experience of installation, they have the opportunity to manipulate masks, at the same time they are used in gathering patient’s opinions a wide range of experience was assured reducing this selection bias. Thus, the results will allow us to share this knowledge with all the actors within this area: physicians, HPs, manufacturers. Moreover, pointing area diagram utilization allows users to share their opinion without the bias of choosing areas by their names, but by their localizations instead. Finally, with an imaging treatment algorithm (free access, open source via the link: https://github.com/BTerzic22/Face_report) this method could be easy to set up and automatized on a larger scale for a fast treatment. The only condition for optimal usage of this algorithm is a good scan quality, without scale deformation and well-defined drawn shapes (dots and crosses). In clinical practice, the usage of this questionnaire could be an interesting monitoring tool of mask tolerance by patients in the CPAP treatment follow-up.

## Conclusion

In summary, this study provided a new methodology to characterize and compare masks of the same type. It also highlighted issues and adaptation difficulties on nasal bridge internal canthus, for facial and nasal masks. This could suggest that both physicians and HPs should costly monitor and pay special attention to the choice of masks, to prevent pressure on nasal bridge and/or leak in the internal canthus area. Moreover, this could be a stake for manufacturers to develop masks that apply low pressure on the nasal bridge and are hermetic on the internal canthus.

## Data Availability

Original data is available on reasonable request to the corresponding author.
